# ame-miR-5119-*Eth* axis modulates larval-pupal transition of western honeybee worker

**DOI:** 10.3389/fphys.2024.1475306

**Published:** 2024-09-27

**Authors:** Shunan Dong, Kunze Li, He Zang, Yuxuan Song, Jing Kang, Ying Chen, Liting Du, Ning Wang, Dafu Chen, Qingming Luo, Tizhen Yan, Rui Guo, Jianfeng Qiu

**Affiliations:** ^1^ College of Bee Science and Biomedicine, Fujian Agriculture and Forestry University, Fuzhou, Fujian, China; ^2^ National and Local United Engineering Laboratory of Natural Biotoxin, Fuzhou, Fujian, China; ^3^ Apitherapy Research Institute of Fujian Province, Fuzhou, Fujian, China; ^4^ Dongguan Maternal and Children Health Hospital, Dongguan, Guangdong, China

**Keywords:** *Apis mellifera*, microRNA, ame-miR-5119, hormone, metamorphosis development

## Abstract

The miRNA plays a key role in the regulation of hormone signaling in insects. The pathways by which miRNAs affect hormone levels are unclear in the honeybee (*Apis mellifera*), an indispensable pollinator in nature. In this study, ame-miR-5119 was overexpressed and knocked down in larvae by feeding mimics and inhibitors, respectively, and we determined that ame-miR-5119 regulates hormone signaling through the target gene ecdysis triggering hormone (*Eth*), which affects the larval-pupal transition of workers. The results showed that ame-miR-5119 with a length of 19 nt targets six genes related to the hormone pathway. We focused on *Eth* and found that ame-miR-5119 and *Eth* exhibited reverse expression patterns during the transition from larval to pupal stages in workers. Dual luciferase assay confirmed the negative regulatory between ame-miR-5119 and *Eth*. Overexpression of ame-miR-5119 decreased the mRNA level of *Eth*, and the Eth receptor (*Ethr*) expression was not significantly affected, but the expression levels of juvenile hormone (JH) pathway related genes *juvenile hormone acid methyltransferase* (*Jhamt*) and *Krüppel homolog* 1 (*Kr-h*1) were significantly reduced. In contrast, knockdown of ame-miR-5119 increased the mRNA level of *Eth*, and the expression of *Ethr*, *Jhamt* and *Kr-h*1 was significantly upregulated. ame-miR-5119 did not affect larval body weight. The number of larvae overexpressing ame-miR-5119 survived in the prepupal stage was lower than that in the control group, and the number of pupations reduced at 11-day-old. The number of larvae that knocked down ame-miR-5119 survived in the prepupal stage was significantly higher than that in the control group, and the number of pupations increased at 11-day-old. These results indicated that ame-miR-5119 negatively regulates the expression of *Eth*, indirectly inhibits the expression of *Ethr*, *Jhamt*, and *Kr*-*h*1, and affects the JH biosynthesis, thereby preventing the metamorphic transition from larva to pupa in worker bees. These findings provide evidence that the miRNA regulation of hormone levels in honey bees.

## 1 Introduction

MicroRNAs (miRNAs) represent a category of intrinsic, diminutive, non-coding RNAs that range from 19 to 24 nucleotides (nt) in length, and play a multifaceted role in various biological processes, including development, metabolism, and DNA damage response (Hrovatin and Kunej, 2018). MiRNAs are highly conserved among various species and usually play a key role as regulatory factors by binding to the 3′-untranslated regions of their target mRNAs (Hrovatin and Kunej, 2018). The functional mechanisms of miRNAs have been extensively elucidated. For instance, the miRNA bantam and miR-14 are involved in modulating organismal growth and cell apoptosis, respectively (Xu et al., 2003; Boulan et al., 2013). Lin-4 and let-7 have been identified to regulate temporal development in *Caenorhabditis elegans* and *Drosophila melanogaster*, respectively (Kaufman and Miska, 2010; Chawla and Sokol, 2011).

There is a direct or indirect relationship between miRNA regulation and hormone pathway in insects. Studies have shown that some miRNAs are components of other systemic signaling pathways in insects, including ecdysone, insulin, stress and immune pathways (Luhur et al., 2013). The steroid hormone 20-hydroxyecdysone (20E) induced an increase in let-7-Complex (let-7, miR-100 and miR-125) in *Drosophila* S2 cells, while juvenile hormone (JH) inhibited let-7 induction in cells treated with 20E (Sempere et al., 2002; 2003). 20E induces upregulation of let-7 in *Bactrocera dorsalis* larvae, whereas let-7 regulates the ecdysone-signalling pathway through the exact dose of E75 gene (Peng et al., 2019). Mosquito miR-1890 modulates the stability of JHA15 mRNA in a tissue-specific manner (Lucas et al., 2015). An increasing number of miRNAs have been confirmed to exert regulatory functions in many ways in the hormone signaling pathways to control the post-transcriptional regulation of insect metamorphosis development (Boulan et al., 2013; Delanoue and Léopold, 2013; Ge et al., 2015).

The Ecdysis Triggering Hormone (ETH), synthesized by Inka cells in the corpora allata, is secreted at precise intervals during the molting cycle. This hormone initiates the molting by activating the central mechanism of molting behavior, thereby playing a pivotal role in triggering and coordinating molting in insect and other arthropod species (Malhotra and Basu, 2023). Prior studies on *Eth* have been documented in various insects, including *Aedes aegypti* (Dai and Adams, 2009), *Drosophila*, and *Bombyx mori*. For instance, the knockout of the *Eth* gene in *Drosophila* results in lethal phenotypes, including respiratory system failure and disrupted sequences of molting behavior (Park et al., 1999). Adams et al. (Adams and Zitnan, 1997) successfully cloned the *Eth* gene in the *B*. *mori* and characterized the natural and hormone-induced behavioral sequences before molting in its larvae, pupae, and adults. However, apart from identifying *Eth* in the global gene expression of honey bees, functional studies of *Eth* have not yet been conducted. ETHR functions as a receptor within the ETH signaling pathway (Malhotra and Basu, 2023). The interaction between ETH and ETHR modulates the activity of JHAMT, which is a crucial enzyme involved in juvenile hormone synthesis, through calcium signaling (Areiza et al., 2014; Shinoda and Itoyama, 2003). JHAMT catalyzes the conversion of JH acids or inactive precursors of JHs to active JHs (Shinoda and Itoyama, 2003). Subsequently, JH binds to the Met/Taiman receptor complex, triggering the expression of primary response gene *Kr-h*1 (Palli, 2023; Lozano et al., 2014). Kr-h1 binds directly to the KBS in the promoters of pupal specifier gene *Br-C* or adult specifier gene *E*93 to inhibit their expression, which in turn prevent pupal metamorphosis or adult metamorphosis (Kayukawa et al., 2017). Studies have shown that components of the JH signaling pathway are highly conserved in insects, including honeybees (Gong et al., 2023; Kayukawa et al., 2017).

Our team conducted transcriptomic investigation of the *A. mellifera* worker 4-, 5-, and 6-day-old larvae based on small RNA-seq (sRNA-seq), during this analytical process, a total of 560 miRNAs, including ame-miR-5119, were identified. In addition, we found that ame-miR-5119 targets the precursor gene of the ecdysis triggering hormone (NM_001142607.1) (Xiong et al., 2018). In this study, we confirmed the expression and sequence of ame-miR-5119 in *A. mellifera* larvaeusing stem-loop RT-PCR and Sanger sequencing. Follow this, we overexpressed and knocked down ame-miR-5119 by feeding specific mimics and inhibitors, respectively; furthermore, we analyzed the effect of ame-miR-5119 overexpression and knockdown on the pupation and longevity of *A. mellifera* worker larvae. Our findingsnot only elucidate the molecular mechanism underlying ame-miR-5119-regulated larval-pupal transition in *A. mellifera* workers, but also provide new insights into the epigenetic modulation of honeybee development.

## 2 Materials and methods

### 2.1 Honey bees

*Apis mellifera* worker larvae were derived from three strong colonies reared in the apiary of the College of Bee Science and Biomedicine, Fujian Agriculture and Forestry University, Fuzhou, China.

### 2.2 Stem-loop RT-PCR and sanger sequencing of ame-miR-5119

We predicted sequence of ame-miR-5119 (5′-TCG​GGG​TCC​TAC​ACT​ACT​C-3′) based on sRNA-sequence (Xiong et al., 2018). Specific stem-loop primers and forward primers (F) as well as universal reverse primers (R) of ame-miR-5119 were designed using DNAMAN software (Table 1) and then synthesized by Sangon Biotech Co., Ltd. (Shanghai, China). The total RNA was isolated from 4-, 5-, and 6-day-old larvae of *A*. *mellifera* workers utilizing an RNA extraction kit (Promega, Madison, WI, United States). Reverse transcription was performed with stem-loop primers using HiScript^®^ III first Strand cDNA Synthesis Kit (Vazyme, Nanjing, China), and the resulting cDNA was served as a template for PCR amplification of ame-miR-5119. The reaction system (20 μL) included 10 μL of 2×Hieff^®^PCR Master Mix (Yeasen, Shanghai, China), 1 μL of forward and reverse primers (2.5 μmol/L), 1 μL of cDNA template, and 7 μL of DEPC water. The reaction conditions were set as follows: predenaturation at 95°C for 5 min, denaturation at 95°C for 50 s, and annealing at 56°C for 30 s and extension at 72°C for 50 s for 34 cycles; and then extended at 72°C for 10 min. PCR amplification was conducted and the amplification product was then detected by 1.5% agarose gel electrophoresis. The expected fragment of 6-day-old larvae was extracted, ligated to the pClone 007 vector (Tsingke, China), and transformed into *E. coli* DH5α competent cells (Tiangen, China). The cloned product was inoculated onto LB agar medium with 50 μg/mL ampicillin, and cultured overnight at 37°C in a biochemical incubator. A single colony was subsequently selected and transferred into LB broth containing 50 μg/mL ampicillin for 12 h of agitational incubation. A sample of the bacterial culture was obtained for PCR analysis, and the PCR-positive samples were sent to Sangon Biotech Co., Ltd. (Shanghai, China) for Sanger sequencing.

**TABLE 1:** Information about primers used in this study.

Name	Sequence (5′-3′)	Purpose
ame-miR-5119-loop	CTC​AAC​TGG​TGT​CGT​GGA​GTC​GGC​AAT​TCA​GTT​GAG​CAG​CCC​CAG	Reverse transcription of ame-miR-5119
ame-miR-5119-F	GCCGAGCTCATCACATC	qPCR detection
ame-miR-5119-R	CTCAACTGGTGTCGTGGA	
Mimic-miR-sense	CUC​AUC​ACA​UCC​UGG​GGC​U	ame-miR-5119 overexpression
Mimic-miR-antisense	CCC​CAG​GAU​GUG​AUG​AGU​U	
Inhibitor-miR	AGC​CCC​AGG​AUG​UGA​UGA​G	ame-miR-5119 knockdown
Mimic-NC-sense	UUC​UCC​GAA​CGU​GUC​ACG​UTT	Negative control for ame-miR-5119 overexpression
Mimic-NC-antisense	ACG​UGA​CAC​GUU​CGG​AGA​ATT	
Inhibitor-NC	CAG​UAC​UUU​UGU​GUA​GUA​CAA	Negative control for ame-miR-5119 knockdown
miR-5119-wt-F	CGT​TAT​CAT​GCC​CGT​GGA​TGA​ATA​TAT​TC	Dual-luciferase assay
miR-5119-wt-R	TCG​AGA​ATA​TAT​TCA​TCC​ACG​GGC​ATG​ATA​ACG​AGC​T	
miR-5119-mut-F	CGT​TAT​CAC​TAA​ATC​TGC​TGA​TAT​ATT​C	
miR-5119-mut-R	TCG​AGA​ATA​TAT​CAG​CAG​ATT​TAG​TGA​TAA​CGA​GCT	
*U*6-F	CCA​GGA​GAT​GAA​GTG​GAT​ACT​C	Internal reference for qPCR of ame-miR-5119
*U*6-R	CTT​GCT​TGA​ACT​GCT​GCT​T	
*actin*-F	CCT​AGC​ACC​ATC​CAC​CAT​GAA	Internal reference for qPCR of target genes
*actin*-R	GAA​GCA​AGA​ATT​GAC​CCA​CCA​A	
*Eth*-F	AGT​GCC​TGC​CTT​CTT​CCT​G	qPCR detection
*Eth*-R	GCC​AAG​ATT​CAG​ACT​GAC​CAT​C	
*Ethr*-F	GCA​TTG​GAA​GCA​GTT​GAA​GAA​G	
*Ethr*-R	GAA​GGT​ACA​GGA​CTC​ACA​GGA​T	
*Jhamt*-F	GTT​GCG​GAC​CTG​GAA​TAG​TTA​C	
*Jhamt*-R	TAA​GCG​TTC​CTC​GTC​GTG​ATA	
*Kr-h*1-F	GCA​TTG​GAA​GCA​GTT​GAA​GAA​G	
*Kr-h*1-R	GAA​GGT​ACA​GGA​CTC​ACA​GGA​T	

### 2.3 Target gene prediction

Miranda (v3.3a) (Betel et al., 2008), RNAhybrid (v2.1.2)+svm_light (v6.01) (Rehmsmeier et al., 2004), and Targeted Scan (v7.0) (Lewis et al., 2005) software were used to predict the target mRNAs of ame-miR-5119, with default parameters for each software. The common mRNAs predicted by three software were used as the target mRNAs of ame-miR-5119. The predicted target mRNAs sequences were then compared with the GO and KEGG databases using the BLAST software to obtain annotation information for the target genes.

### 2.4 Dual-luciferase assay

The potential binding sites between *Eth* and ame-miR-5119 was predicted using the RNA hybrid software (v.2.1.2). Specific primers for the aforementioned binding sites were designed (Table 1), followed by the use of PCR amplification. The amplified fragments were then cloned into pmirGLO vectors and named miR-5119-wt. Concurrently, the mutant sequences of the above binding sites were designed and synthesized, and then cloned into pmirGLO vectors, designated as miR-5119-mut. The bacterial fluids were sent to Sangon Biotech Co., Ltd. (Shanghai, China) for Sanger sequencing, and reverse transcription using reverse transcription using reverse transcription using reverse transcription using those samples that were sequenced correctly were transferred to a fresh liquid LB medium. Plasmids were extracted using an Endotoxin Removal Plasmid Extraction Kit (Beijing Total Gold Biotechnology Co., Ltd., Beijing, China).

The HEK-293T cells placed into a 37°C incubator for 24 h to reach a cell density of 90%–95%. Cell transfection experiments were carried out by following the instructions for the Hieff Trans™ Liposomal Nucleic Acid Transfection Reagent (Shanghai Yeasen Biotechnology Co., Ltd., Shanghai, China), and 4 transfection groups were set up at the same time: 1) mimic-miR-5119 co-transfected with pmirGLO-*Eth*-miR-5119-wt; 2) mimic-NC co-transfected with pmirGLO-*Eth*-miR-5119-wt; 3) mimic-miR-5119 co-transfected with pmirGLO-*Eth*-miR-5119-mut; 4) mimic-NC co-transfected with pmirGLO-*Eth*-miR-5119-mut. After the transfection was completed, the cell culture plates were incubated in a 37°C incubator for 24 h. Further, the viability of firefly fluoresceinase and Renilla fluoresceinase was detected on a dual-luciferase assay reporter system (Promega, Madison, WI, United States) using a dual-luciferase detection kit (Shanghai Yeasen Biotech Co., Ltd.), and the relative expression folds were obtained by calculating the ratio of firefly fluoresceinase/Renilla fluoresceinase. Following the method described by Matsumura et al. (2022) and Wang et al. (2023). This experiment was repeated in triplicate.

### 2.5 Real-time fluorescence quantitative polymerase chain reaction (RT-qPCR)

Specific primers of *genes* were designed using Primer Premier 6 software (Table 1) and then synthesized by Sangon Biotech Co., Ltd. (Shanghai, China). Total RNA treated with DNase from *A. mellifera* worker larva at 3-day-old (larval stage), 7-day-old and 8-day-old (prepupal stage), 12-day-old (pupal stage) by respectively extracted using an RNA extraction kit (Promega, Madison, WI, United States) (n = 3), and subsequently divided into two portions for reversing transcription using the HiScript^®^ III first Strand cDNA Synthesis Kit (Vazyme, Nanjing, China). One portion was subjected to reverse transcription with stem-loop primers, and the resulting cDNA was used as templates for RT-qPCR detection of ame-miR-5119; the other portion was reverse transcribed using a 1:1 ratio of Oligo (dt) and Random primers, and the resulting cDNA was used as templates for RT-qPCR of other genes. Following the instructions of the Hifair^®^ qPCR SYBR Green Master Mix (Low Rox Plus) kit (Yeasen, Shanghai, China), RT-qPCR was conducted on a QuantStudio 3 fluorescent quantitative PCR system (ABI Company, Tampa, FL, United States). *U*6 (GenBank ID: LOC725641) was used as the internal reference of ame-miR-5119 and *actin* (GenBank ID: NM001185145) was used as the internal reference of *Eth, Ethr, Jhamt,* and *Kr-h*1 genes. The reaction system (20 μL) included 10 μL of Hifair^®^ qPCR SYBR Green Master Mix (Low Rox Plus) (Yeasen, Shanghai, China), 1 μL of forward and reverse primers (2.5 μmol/L), 1 μL of cDNA template, and 7 μL of DEPC water. The reaction conditions were established as follows: predenaturation at 95°C for 3 min, denaturation at 95°C for 10 s, and annealing and extension at 60°C for 30 s for a total of 42 cycles; the melting curve program was set to the default setting of the system. Three individuals are collected and mixed together as a single biological sample, each of biological sample underwent three technical replicates, and a total of three independent biological samples. The relative expression level of ame-miR-5119 was calculated using the 2^−ΔΔCT^ method (Livak and Schmittgen, 2001).

### 2.6 Overexpression and knockdown of ame-miR-5119 in *Apis mellifera* larva

According to the method described by Wang et al. (Wang et al., 2019), ame-miR-5119 mimics (mimic-miR-5119) and inhibitors (inhibitor -miR-5119) as well as corresponding negative controls (mimic-NC and inhibitor-NC) were designed using Dharmacon (Lafayette, Colorado, United States) software (Table 1) and synthesized by GenePharma (Shanghai, China). According to previously described method (Guo et al., 2018), 2-day-old larvae of *A. mellifera* were carefully transferred to 6-well culture plates containing 800 μL of artificial diet, with 40 larvae per well, and the plates were subsequently placed in a constant temperature and humidity incubator (35°C ± 0.5°C, RH 90%) for 24 h. 3-day-old larvae were transferred to 48-well culture plates, each larva was fed 50 μL of artificial diet containing mimic-miR-5119, Mimic-NC, inhibitor-ame-miR-5119 or Inhibitor-NC (40 pmol/g). Every 24 h thereafter, 50 μL of diet was added to each well until 6-day-old (Stopped eating at 7-day-old). 4-, 5-, and 6-day-old larvae (Three biological samples, n = 3) were collected into sterile and RNA-free microcentrifuge tubes. They were immediately frozen in liquid nitrogen and subsequently stored at −80°C for future use. RT-qPCR was performed to evaluate the effects of overexpression and knockdown of ame-miR-5119 from 4-, 5-, and 6-day-old larvae.

### 2.7 Measurement of body weight

Based on the method described by Borsuk et al. (2017), 4-, 5-, and 6-day-old larvae in each group (n = 3 for each age) were rinsed three times with phosphate buffer saline (PBS) to remove diet residue on the body surface, followed by absorption of the fluid on the larval body surface with clean filter paper. The larvae were weighed using FA2004 electronic scales (Shanghai Shunyu Hengping, Shanghai, China).

### 2.8 Statistics of pupation and survival rates

Following the method described in Section 2.5, 3-day-old larvae were transferred to 48-well culture plates and fed. Each larva was fed 50 μL of artificial diet containing mimic-miR-5119, mimic-NC, inhibitor-miR-5119 or inhibitor-NC (40 pmol/g, n = 72). Every 24 h thereafter, 50 μL of diet was added to each well until day 6-day-old. The number of pupation and survival of larvae were continuously counted from 3-day-old from 12-day-old. The number of pupation and survival of larvae were continuously observed and counted. Furthermore, statistics of pupation and survival rates were visualized using Graph Pad Prism 10 software.

### 2.9 Statistical analysis

GraphPad Prism version 10 (GraphPad, San Diego, CA, United States) was used for graph construction and statistical analysis. Data are presented as the mean ± SD. Statistical analysis was performed using the one-way ANOVA or Two-way ANOVA, followed by Tukey’s multiple comparisons.

## 3 Results

### 3.1 Molecular verification and target prediction of ame-miR-5119

**FIGURE 1:**
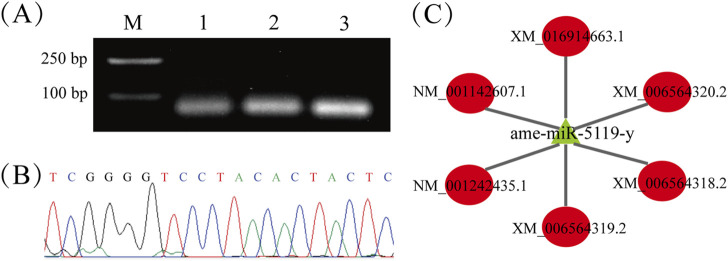
Molecular validation and regulatory network of ame-miR-5119. **(A)** Detection of the amplification products from ame-miR-5119 by stem-loop RT-PCR, Lane M: DNA marker, Lane1-3: 4-, 5-, and 6-day-old larvae; **(B)** Peak diagram of the signal fragment amplified from stem-loop RT-PCR; **(C)** Regulatory network between ame-miR-5119 and 6 target genes. The mRNAs XM_006564318.2, XM_006564319.2, XM_006564320.2 and XM_016914663.1 are *ecdysone-induced protein* 75 gene (E75); The mRNA NM_001242435.1 are *ecdysoneless* gene (*ecd*); NM_001142607.1 are *Eth* gene.

Stem-loop RT-PCR showed that the expected fragment (approximately 100 bp) was amplified from the 4-, 5-, and 6-day-old larvae (Figure 1A). Sanger sequencing obtained a 19 nt sequence that was consistent with the ame-miR-5119 sequence predicted by transcriptome data (Figure 1B). The prediction results of the target mRNA regulated by ame-mir-5119 showed that the top six target mRNAs were all hormone signaling pathway related genes, including *ecdysone*-*induced protein* 75 gene (*E*75), *ecdysoneless* gene (*ecd*,), and *Eth* (Figure 1C).

### 3.2 ame-miR-5119 negatively regulates the expression of *Eth*

**FIGURE 2:**
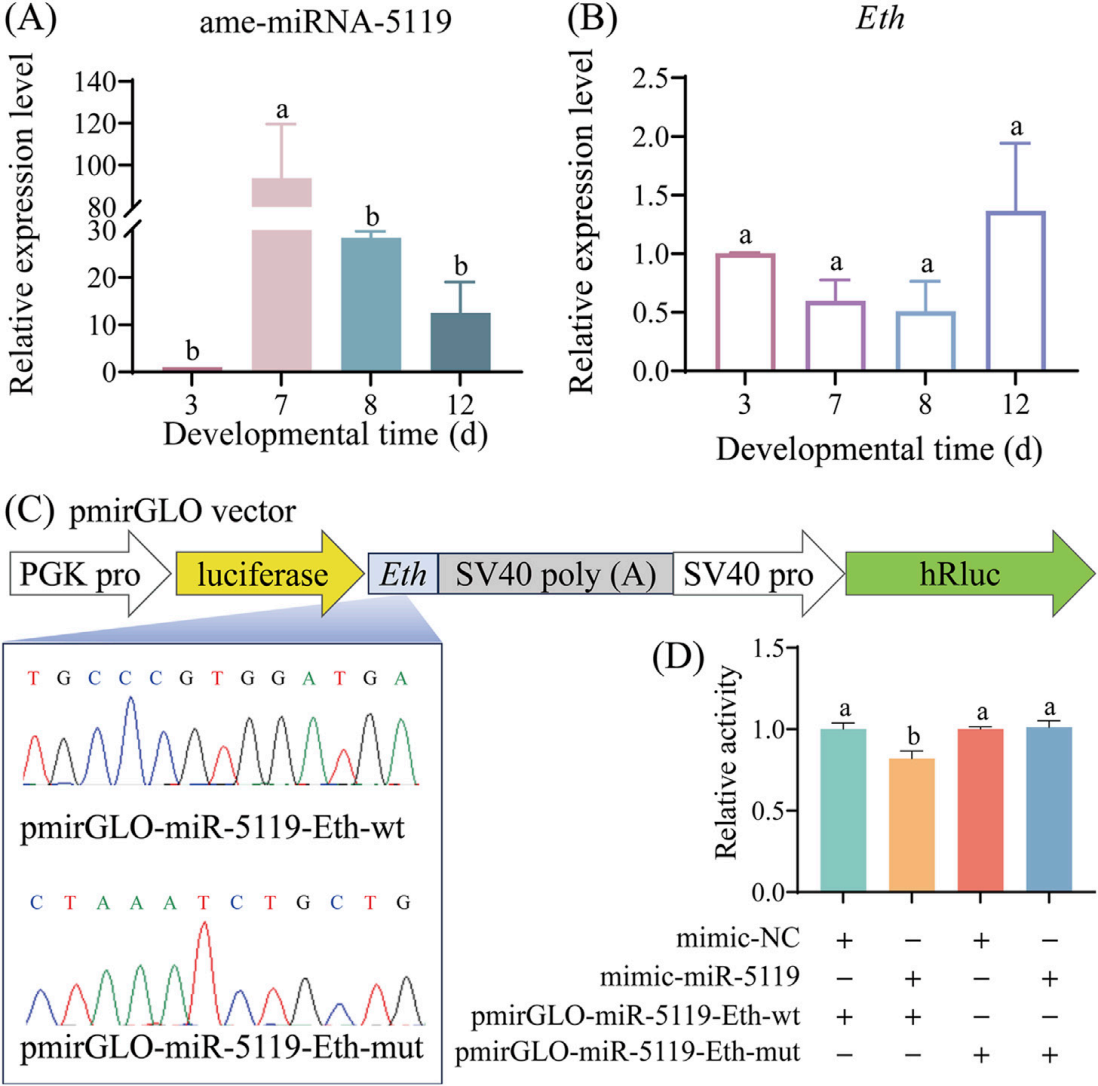
Expression pattern determination and binding relationship confirmation of ame-miR-5119 and *Eth*. Relative expression levels of ame-miR-5119 **(A)** and *Eth* gene **(B)** in *A. mellifera* worker larva (3-day-old), prepupa (7-day-old and 8-day-old), and pupa (12-day-old). Two-way ANOVA, Tukey’s multiple comparisons, n = 3. **(C)** pmirGLO vector construction model for dual luciferase analysis, and Sanger sequencing of the *Eth* mutated binding sites. **(D)** Dual-luciferase reporter assay of the binding relationship between ame-miR-5119 and *Eth*. One-way ANOVA, n = 3. Different letters above bars indicate groups that are statistically significant (*p* < 0.05).

The temporal expression of ame-miR-5119 and *Eth* was detected, and four typical development stages were selected: the middle time of larval stage (3-day-old), prepupa preparation stage (7-day-old), prepupal stage (8-day-old) and pupal stage (12-day-old). The results revealed that the expression level of ame-miR-5119 was significantly higher at the 7-day-old compared to other developmental stages, followed by the 8-day-old, and a lower expression level observed at the 3-day-old and 12-day-old (Figure 2A). Conversely, the expression level of *Eth* was lowest at 7-day-old and 8-day-old, and elevated at 3-day-old and 12-day-old (Figure 2B). As presented in Figure 2C, recombinant plasmids pmirGLO-miR-5119-*Eth*-wt and pmirGLO-miR-5119-*Eth*-mut for dual luciferase assay were successfully constructed. Dual luciferase assay showed that ame-miR-5119 significantly inhibited the transcription of *Eth* compared to the mimic-NC group. Mutating the binding target site in *Eth* (*Eth*-mut), the expression of *Eth* was not affected by ame-miR-5119 (Figure 2D). These results together indicate th at ame-miR-5119 targets *Eth* and negatively regulates the expression of *Eth*.

**FIGURE 3:**
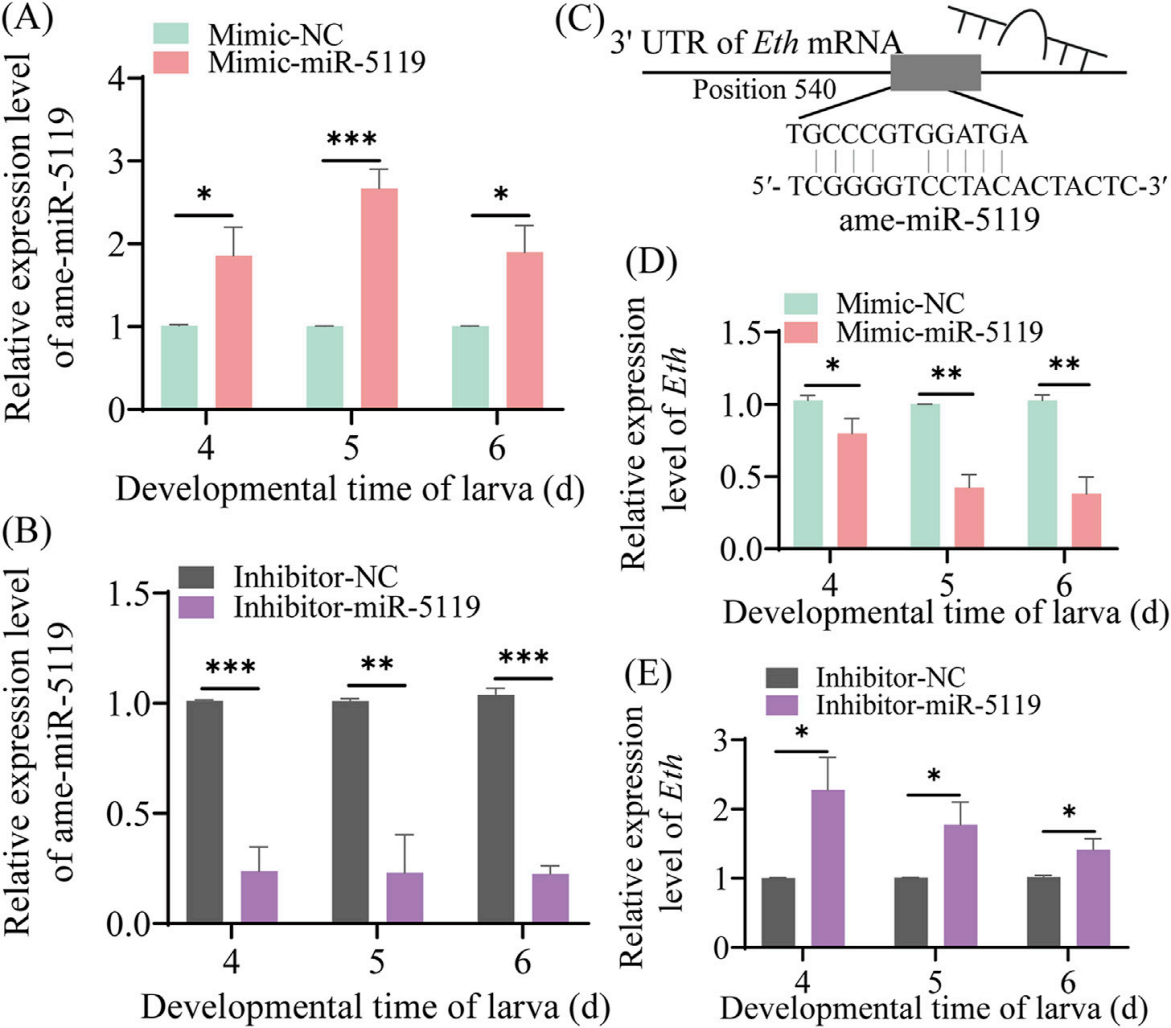
Effect of ame-miR-5119 overexpression and knockdown on *Eth* expression in *A. mellifera* worker larvae. **(A, B)** Relative expression level of ame-miR-5119 in the larva after feeding mimic-miR-5119 and inhibitior-miR-5119. **(C)** A schematic diagram of the target binding site between ame-miR-5119 and *Eth*. **(D, E)** RT-qPCR detection of the expression level of *Eth* in the 4-, 5-, and 6-day-old larvae after ame-miR-5119 overexpression or knockdown. Two-way ANOVA, Tukey’s multiple comparisons, n = 3. *, *p* < 0.05; **, *p* < 0.01; ***, *p* < 0.001; ns, non-significant.

The results of RT-qPCR detection showed that the expression level of ame-miR-5119 was significantly upregulated in the 4-, 5-, and 6-day-old larvae in the mimic-miR-5119 group in comparison with that in the mimic-NC group (*p* < 0.05) (Figure 3A). Comparatively, the expression level of ame-miR-5119 was significantly downregulated in 4-, 5-, and 6-day-old larvae in the inhibitor-miR-5119 group as compared to that in the inhibitor-NC group (*p* < 0.05) (Figure 3B). The results indicated that effective overexpression and knockdown of ame-miR-5119 in the *A. mellifera* larvae were achieved by feeding specific mimic and inhibitor. As presented in Figure 3C, ame-miR-5119 had reverse complementary binding target to *Eth* mRNA. Subsequent analyses revealed that the *Eth* expression levels of 4-day-old, 5-day-old, and 6-day-old larvae in the mimic-miR-5119 group were significantly elevated compared to those in the mimic-NC group (*p* < 0.05) (Figure 3D). Similarly, the *Eth* expression levels of larvae at these same ages in inhibitor-miR-5119 group were markedly reduced relative to the inhibitor-NC group (*p* < 0.05) (Figure 3E).

### 3.3 ame-miR-5119 regulates JH pathway by targeting *Eth* in *A*. *mellifera* worker larvae

**FIGURE 4:**
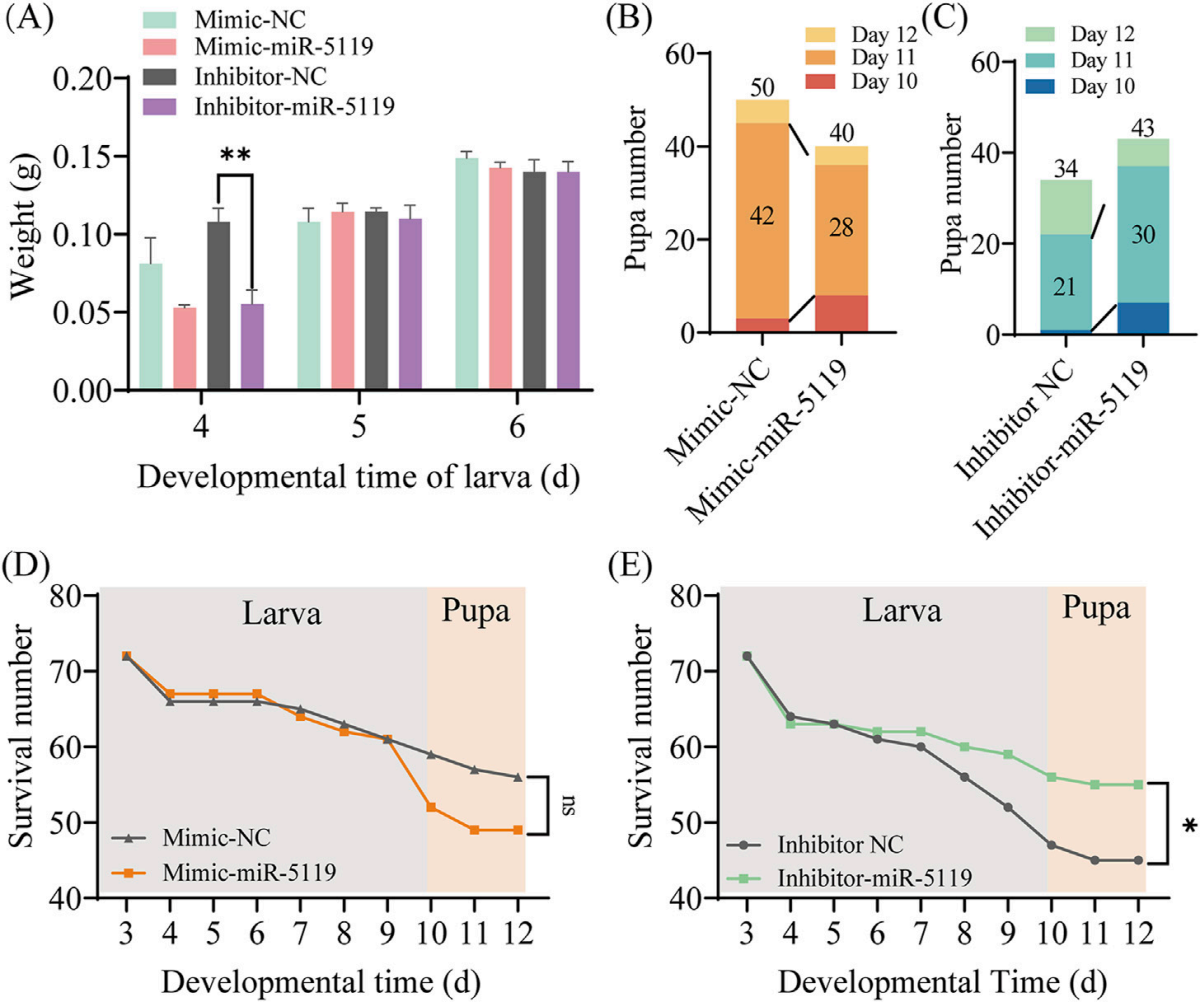
Effect of overexpression and knockdown of ame-miR-5119 on the (A) *mellifera* larvae. **(A)** The body weight of *A. mellifera* worker after ame-miR-5119 overexpression and knockdown. Multiple *t* tests, n = 3, **, *p* < 0.01. **(B, C)** The number of pupae after ame-miR-5119 overexpression and knockdown (n = 34–50). **(D, E)** The survival number of pupae after ame-miR-5119 overexpression and knockdown (n = 72). Two-way ANOVA, Tukey’s multiple comparisons. *, *p* < 0.05; ns, non-significant.

The development of worker larvae was investigated after overexpression or knockdown of ame-miR-5119, the results were indicative of a gradual increase in larval body weight with rearing time. Overexpression or knockdown of ame-miR-5119 did not affect body weight, only the weight of larvae in the inhibitor-miR-5119 group was significantly lower than that of larvae in the inhibitor-NC group at 4-day-old (Figure 4A). Larval pupation was mainly observed on day 10 to day 12, the results suggested that the number of pupate in the mimic-miR-5119 group was remarkedly lower than that of pupae in the mimic-NC group at day 11, and the total number of pupae also decreased (Figure 4B). In contrast, the number of pupae in the inhibitor-miR-5119 group was obviously higher than that in inhibitor-NC group on day 11, and the total number of pupae as well increased (Figure 4C). Additionally, the survival of larvae after overexpression or knockdown of mimic-miR-5119 was analyzed, the results demonstrated that the survival number of individuals in the mimic-miR-5119 group did not differ from that of the mimic-NC group from day 3 to day 9, but that of individuals in the mimic-miR-5119 group decreased remarkedly after pupation at day 10 (Figure 4D). However, the survival number of individuals in the inhibitor-miR-5119 group was significantly higher than that in the inhibitor-NC group. Notably, the number of deaths in the pupal stage was lower in the inhibitor-miR-5119 group (Figure 4E).

The expression of *Ethr*, the receptor gene of *Eth*, was detected by RT-qPCR. The results revealed no significant difference in the expression level of *Ethr* following mimic-miR-5119 overexpression. However, knockdown of ame-miR-5119 resulted in a decrease in *Ethr* expression. Further investigation of the expression of JH synthesis gene *Jhamt* and JH receptor gene *Kr-h*1 downstream of *Eth* was conducted, the results showed that the expression levels of these two genes were significantly decreased following overexpression of ame-miR-5119 while significantly increased after ame-miR-5119 knockdown. These results indicated that ame-miR-5119 affects the expression of downstream *Jhamt* and *Kr-h*1 genes through target gene *Eth*, which in turn affects the metamorphosis of honeybee from larva to pupa.

## 4 Discussion

Due to a lack of bioinformatic analysis and functional investigation, the role of ame-miR-5119 in *A. mellifera* was previously completely unknown. In the present study, the expression and sequence of ame-miR-5119 were confirmed by stem-loop RT-PCR and Sanger sequencing, providing a basis for continuous investigation of the regulatory function of ame-miR-5119 in various physiological and pathological processes. In addition, we observed that the expression patterns of ame-miR-5119 and *Eth* were opposite at different developmental stages (Figure 2), suggestive of a potential regulatory relationship between them. Furthermore, through the system established by our group (Zhang Q. et al., 2022; Wu et al., 2023), effective overexpression and knockdown of ame-miR-5119 were observed following feeding specific mimics and inhibitors (Figure 3). Collectively, the results from this study and our previous work indicated that overexpression and knockdown were successfully achieved in the *A. mellifera* larvae by feeding mimics and inhibitors, providing a reliable platform for functional studies on larval miRNAs in bees.

In our previous study, the potential targeting relationship between ame-miR-5119 and the *Eth* gene was predicted using bioinformatics (Xiong et al., 2018). Here, dual luciferase assay showed that ame-miR-5119 targeted *Eth*. RT-qPCR results indicated that ame-miR-5119 negatively regulates the expression of *Eth* (Figure 3). Together, these results verified the targeting and negative regulatory relationship between ame-miR-5119 and *Eth*.

**FIGURE 5:**
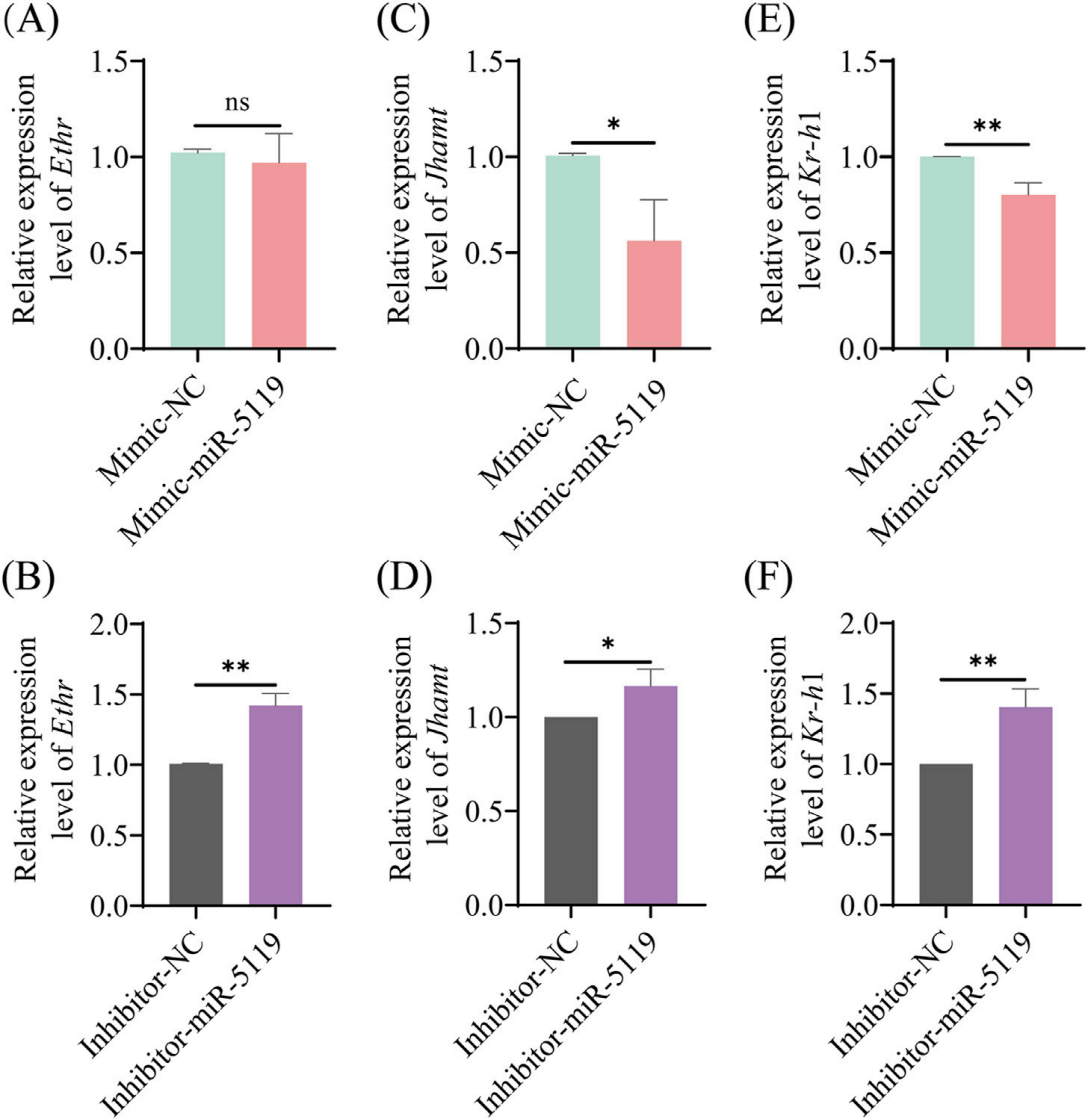
Relative expression levels of *Ethr* **(A, B)**, *Jhamt* **(C, D)** and *Kr-h*1 **(E, F)** genes in *A. mellifera* worker larvae after ame-miR-5119 overexpression and knockdown. Unpaired *t*-test, n = 3. *, *p* < 0.05; **, *p* < 0.01; ***, *p* < 0.001; ns, non-significant.

Genes encoding Eth and ecdysis that triggers hormone receptors (Ethr, Ethr-A, and Ethr-B) were identified and well-characterized in various holometabolous insects, such as *B*. *mori* (Zitnan et al., 2002)*, Tribolium castaneum* (Arakane et al., 2008) and *Schistocerca gregaria* (Lenaerts et al., 2017). Areiza et al. found that silencing *Ethr*s by RNA interference (RNAi) in pupa resulted in reduced juvenile hormone (JH) synthesis (Areiza et al., 2014). *Eth* also contributes toJH biosynthesis and the activation of the key biosynthetic enzyme Jhamt of the JH biosynthetic pathway through the mobilization of Ca^2+^ from intracellular and extracellular stores, thereby ensuring proper developmental timing in pharate adult insects (Areiza et al., 2014). The receptor complex Met/Tai mediates the transcription of *Kr-h*1 by binding to the E-box or E-box-like DNA elements within the promoter region under the influence of the JH, playing a pivotal role in the regulation of insect metamorphosis (Campli et al., 2024; Li, 2020). The expression level of *Jhamt* at 6-day-old larvae was significantly downregulated and that of *Kr-h*1 was significantly downregulated after ame-miR-5119 overexpression (Figure 5). Our findings substantiate the role of ame-miR-5119 as a negative regulator of *Eth* expression during the larval stage. The metamorphic development of insects is regulated by JH. *Jhamt* serves as a pivotal enzyme gene in the biosynthetic pathway of JH, encoding the juvenile hormone acid methyltransferase. ETHR and Kr-h1 influence the synthetase and downstream signal transduction of JH, respectively. This regulatory pathway underscores the intricate interplay between miRNAs and hormone signaling in determining the developmental trajectory of honeybee larvae. As a holometabolous insect, *A. mellifera* undergoes comprehensive tissue remodeling and transformation within its body during the prepupal stage and even late larval phase. The prepupal development is remarkably rapid, initiating the generation of adult head and thorax structures, even though the abdomen has not yet retracted and separated from the thorax. compound eyes has already begun, indicating the significance of this progress in the metamorphic transition towards adulthood (Marco Antonio and Hartfelder, 2017). The titer of JH is the key to regulate the development of larval metamorphosis (Rembold, 1987). Studies have shown that miRNAs were engaged in the regulation of insect physiology including body weight (Fang et al., 2022; Zhang K. Y. et al., 2022). In this study, the total number of pupations in the mimic-NC group, which consisted of 10- to 12-day-old pupae, surpassed that in the mimic-miR-5119 group. The total number of pupae in the inhibitor-NC group was lower than that in the inhibitor-miR-5119 group, with the disparity being particularly pronounced at 12 days of age (Figure 4). This suggests that the impact of ame-miR-5119 overexpression and knockdown on pupation may be linked to alterations in JH titer.

Small Inka cells were identified as sites for *Eth* expression and are scattered throughout the tracheal system in hemimetabolous and some holometabolous insects (Malhotra and Basu, 2023). In the study of Kim et al. (2018) tracheal air-filling defects were observed upon RNAi silencing of Eth receptors in Kinin neurons, and these flies showed low survival rates during development, indicating that *Eth* modulated tracheal air-filling via downstream Kinin signaling. Additionally, survival rate statistics demonstrated that larvae in the inhibitor-miR-5119 group consistently exhibited higher average survival rates than those in the inhibitor-NC group, and these rates surpassed the average survival rate of the mimic-miR-5119 group after day 9 (Figure 4). This indicates that the negative regulation of *Eth* expression by ame-miR-5119 is likely to affect the larval mortality at the late stage of larval development.

**FIGURE 6:**
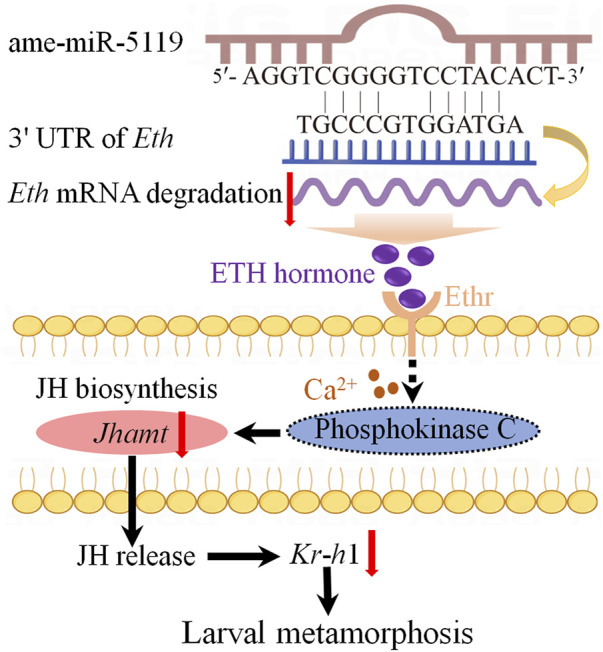
A speculated model of ame-miR-5119 regulating larval metamorphosis through *Eth*. The ame-miR-5119 negatively regulates the expression of *Eth*, indirectly inhibits the expression of *Ethr*, *Jhamt*, and *Kr*-*h*1, affects the JH biosynthesis, thereby prevents the metamorphosis of *A. mellifera* larvae.

Combined with previous studies (Areiza et al., 2014), we have summarized the potential mechanisms by which ame-miR-5119 influences larval metamorphosis. Specifically, ame-miR-5119 exerts a negative regulatory effect on *Eth* expression and reduces ETH synthesis. Subsequently, ETH binds to the ETHR receptor and acts on phosphokinase C through calcium signaling, thereby modulating JH biosynthesis. Ultimately, these processes collectively impact the metamorphosis and survival rate of *A. mellifera* larvae (Figure 6).

## Data Availability

The data analyzed in this study is subject to the following licenses/restrictions: Datasets publicly are unavailable. Requests to access these datasets should be directed to RG, ruiguo@fafu.edu.cn.
